# Impact of *Spirulina platensis* biomass on the viability of *Lactobacillus delbrueckii* subsp. *bulgaricus* strain during the freeze-drying process

**DOI:** 10.5114/bta.2024.139751

**Published:** 2024-06-25

**Authors:** Ivo Ganchev

**Affiliations:** Trakia University of Stara Zagora, Faculty of Technics and Technologies of Yambol, Yambol, Bulgaria

**Keywords:** *Lactobacillus delbrueckii* subsp, *bulgaricus*, *Spirulina platensis* biomass, freeze-drying, storage, cell viability, cryotoloerance

## Abstract

In this work, we evaluated the protective capacity of *Spirulina platensis* biomass in preserving *Lactobacillus delbrueckii* subsp. *bulgaricus* WDCM 00102. The *L. bulgaricus* strain was freeze-dried in the presence of *S. platensis* biomass and the freeze-dried samples were then stored at 5 and 25°C for varying periods. Subsequently, the samples were rehydrated and bacterial plate counts were determined. The results indicate that a concentration of 12% *S. platensis* biomass was highly effective in preserving *L. bulgaricus*. Commercial products with higher *S. platensis* biomass content exhibited greater protective capacity. While *S. platensis* biomass is well-known for its prebiotic properties, its protective role has not been previously reported or thoroughly explored. This study demonstrates the protective capacity of *S. platensis* biomass in preserving *L. bulgaricus*, a strain particularly sensitive to preservation processes.

## Introduction

Lactic acid bacteria and bifidobacteria are widely used probiotics in functional foods. Their preservation typically involves freezing or freeze-drying to maintain the viability and key technological properties such as acidification activity, aroma production, texture formation, and probiotic properties (Górska et al., 2019). These bacteria produce lactic acid as the primary product of glucose and also release growth-inhibiting substances like bacteriocins, hydrogen peroxide, diacyls, etc., which prevent the proliferation of food spoilage bacteria and pathogens (Mokoena, 2017).

The probiotic effects of these microorganisms include preventing constipation in the elderly, diarrhea prevention, immune system stimulation (Beganovic et al., 2011), alleviation of lactose intolerance, reduction of blood cholesterol levels, and cancer prevention (Ghosh et al., 2019). Additionally, probiotics offer protection against various opportunistic human pathogens such as *Helicobacter pylori*, *Salmonella enterica* serovar *Typhimurium*, *Bacteroides fragilis*, *Bacteroides thetaiotaomicron*, *Pseudomonas aeruginosa*, and *Clostridium difficile* (Mays and Nair, 2018).

One cancer-prevention mechanism involving probiotic bacteria, primarily *Lactobacillus* and *Bifidobacillus* strains, may be linked to their ability to bind to and degrade potential carcinogens. Mutagenic compounds associated with an increased risk of colon cancer are often present in unhealthy foods, especially fried meats. Human volunteers who consumed *Lactobacillus* strains experienced reduced mutagenic effects from diets rich in cooked meat, leading to decreased urinary and fecal excretion of heterocyclic aromatic amines (HAAs) such as 3-amino-1-methyl-5H-pyrido (4,3-β) indole, nitrosamines, mycotoxins, polycyclic aromatic hydrocarbons (PAHs), and phthalic acid esters (PAEs) (Górska et al., 2019).

Lactic acid bacteria utilize carbohydrate fermentation to generate energy, using endogenous carbon sources as the final electron acceptor instead of oxygen (Mokoena, 2017). Prebiotics are closely linked to probiotics as they are defined as fermentable substrates that selectively stimulate the growth and/or activity of intestinal bacteria due to their enzymatic activities (e.g., α-glucosidases and β-glucosidases, α-galactosidases and β-galactosidases, and β-fructofuranosidase), contributing to health and well-being (Tabasco et al., 2014).

Potential prebiotic oligosaccharides can be classified based on their chemical constituents and degree of polymerization (d.p.), including manno-oligosaccharides, pectic-oligosaccharides, soybean-oligosaccharides, isomaltooligosaccharides, xylooligosaccharides, and lactulose (Barroso et al., 2016). Fructooligosaccharides and galactooligosaccharides are resistant to gastric acid and are not substrates for hydrolytic enzymes in the upper digestive tract. They are also stable at high temperatures in acidic environments, making them particularly interesting to the food and beverage industry for their prebiotic properties and use as sweeteners, especially in confectionery, acidic beverages, and fermented milk (Mokoena, 2017).

Oligosaccharides typically occur in natural sources as mixtures of compounds with varying degrees of polymerization (Macfarlane et al., 2008). Macroalgae are considered a rich source of sulfated polysaccharides. For example, extracted polysaccharides from Ulva spp. (12% of the algal dry weight) have been reported to contain 16% sulfate and 15–19% uronic acids (Hubálek, 2003). Brown algae contain alginic acid as an anionic polysaccharide, with content varying among species, such as *Ascophylum nodosum* – 22–30%, *Laminaria digitata* – 25–44%, *L. digitata* – 35–47%, *Laminaria hyperborea* – 7–33%, *L. hyperborea –* 25–38%. Brown algae also contain fucoidan (5–20%), with about 40% of it being sulfate esters. Laminarin can constitute up to 35% of the dried weight of seaweed. Most red algal polysaccharides are galactans with alternating α-(1,3) and β-(1,4) links. Uronic acids have been reported in red seaweed polysaccharides from species like *Palmaria decipiens*, *Pterocladiella capillacea*, and Hypnea spp. (O’Sullivan et al., 2010).

These relatively high molecular weight polysaccharides are rich in hydroxyl (OH) groups, making them hydrophilic. They are known to form intrachain H-bond networks, imparting stiffness and rigidity, which makes them suitable as thickeners. The regularity of their structures also facilitates interaction with external ions and interchain H-bonding, leading to processes like gelation (O’Sullivan et al., 2010; Rastoll et al., 2013).

Spirulina has been used for many years as a human food additive due to its high protein content and nutritional value (Rastoll et al., 2013). In previous studies, *Lactococcus lactis*, *Streptococcus thermophilus*, *Lactobacillus casei*, *Lactobacillus acidophilus*, and *Lactobacillus bulgaricus* were grown in rich MRS and minimal saline medium in the presence and absence of extracellular products obtained from a late log phase culture of *Spirulina platensis* (Santos et al., 2014). Both MRS and M17 media significantly promoted the growth of the lactic acid bacteria tested (Parada et al., 1998). *S. platensis* is a planktonic, filamentous blue-green algae found in various freshwater environments, including ponds, lakes, and rivers (O’Sullivan et al., 2010). Spirulina platensis contains approximately 13.6% carbohydrates, including glucose, rhamnose, mannose, xylose, and galactose. *S. platensis* biomass is known to stimulate bacterial growth, and studies suggest that the algae consume nitrogen from the growth medium and release extracellular carbohydrates and other growth substances that may stimulate the growth of *Lactobacillus* strains (and other lactic acid-producing strains) (Santos et al., 2014).

A combination of probiotics and prebiotics forms a synbiotic, which can stimulate and increase the survival of probiotic and autochthonous-specific strains in the intestinal tract (Gourbeyre et al., 2011), enhance healthy colonic microbiota composition, and improve the survival of bacteria crossing the upper part of the gastrointestinal tract, thereby enhancing their effects in the large bowel (Jain et al., 2014). The use of synbiotic products, where probiotics are supplemented with compounds like galacto-oligosaccharides and fricto-oligisaccharides, can open up new commercial applications, as these compounds exert both prebiotic and protective effects (Tymczyszyn et al., 2011).

The crucial role of lactic acid bacteria as starters in the production of dairy and pharmaceutical products underscores the need for appropriate preservation processes. Freeze-drying is the most commonly used technique for drying microorganisms, based on sublimation occurring in three phases involving a freezing step followed by two-stage drying processes under high vacuum (Novoselova and Stoyanova, 2021). However, this process can lead to cellular damage due to the formation of crystals and osmotic stresses (Rault et al., 2010). Several factors can reduce the viability of probiotics, including matrix acidity, oxygen levels in products, the presence of other lactic acid bacteria, and sensitivity to metabolites produced by competing bacteria (Putta et al., 2018). Adaptive stress responses in probiotics involve alterations in various physiological features and structural cell components (De Angelis et al., 2004).

For the acid tolerance response, mechanisms linked to pH homeostasis include the proton-translocating F1F0-ATPase, modification of cell membrane properties, increase in cytoplasmic alkalinity through the activity of enzymes like arginine deaminase, urease, and glutamine decarboxylase, and production of stress proteins (Lapsiri et al., 2012). Cellular targets for damage during freeze-drying include membranes, nucleic acids, and certain enzymes (Grattepanche et al., 2013). To protect cells against such damage during freeze-drying, a wide range of lyoprotectants, such as skim milk, can be added to the drying media. Polyhydroxylated compounds like sucrose and trehalose are commonly used as protecting agents during bacterial preservation (Novoselova and Stoyanova, 2021).

Sugars can replace water molecules during dehydration by forming hydrogen bonds around the polar and charged groups present in phospholipid membranes and proteins, thereby stabilizing their native structure in the absence of water (Rault et al., 2010). This process inhibits oxidative cell damage caused by the scavenging of free radicals (Tymczyszyn et al., 2012). High survival rates of lactic acid bacteria in freeze-dried form have been achieved by combining sugars and sugar derivatives with salt ions or complex media, including skim milk (Ying et al., 2012), which prevents intracellular ice formation within the bacteria, thus maintaining their viability when frozen (Grattepanche et al., 2013).

Probiotics exhibit high stability in dairy products compared to nondairy products, a factor dependent on the selection of robust bacterial strains capable of withstanding harsh environmental conditions and the type of prebiotics used (Lapsiri et al., 2012). Cryopreservation using polysaccharide- or protein-based systems has proven more effective in protecting bacteria during freeze-drying and storage compared to traditional cryoprotection methods. When combined with the effects of polysaccharides, some of which are used as prebiotics, this approach allows for a suitable delivery system that protects probiotic bacteria and may result in a synbiotic formulation (Heidebach et al., 2010). While cryopreservation is common in microorganisms, especially bacteria and fungi, there is limited information available on its application to microalgae (Rastoll et al., 2013). Microalgae are photosynthetic microorganisms capable of producing various metabolites, including lipids, proteins, carbohydrates, and pigments (Silva et al., 2016). Some combinations of protective agents, including skim milk, disaccharides (such as sucrose and trehalose), proteins (e.g., bovine serum albumin), and amino acids, have been tested on Lactobacillus strains for use as probiotics (Haiping et al., 2019).

The objective of this study was to assess the effectiveness of *S. platensis* biomass in the recovery of *Lactobacillus delbrueckii* subsp. *bulgaricus* after freeze-drying. *L. bulgaricus* was chosen due to its sensitivity to temperatures below 0°C (Bauer et al., 2012). Starters of *L. bulgaricus*, typically preserved by freezing, freeze-drying, and spray drying, are widely used in dairy product production (Santos et al., 2014; Tymczyszyn et al., 2012). To comprehensively evaluate the protective properties of *S. platensis* biomass, the acid production kinetics and survival after storage at various temperatures were determined.

## Materials and methods

### Spirulina platensis biomass

*S. platensis* powder was obtained from Simbiotex Ltd., Sofia, Bulgaria. The powder was rehydrated in Zarrouk medium at 28°C in an orbital shaker at 120 rpm, exposed to 85 μmol photons m^-2^s^-1^ of cool-white fluorescent light with a 16/8 h light/dark cycle. The algae were grown photoautotrophically in 2 l Erlenmeyer flasks containing 1.6 l of Zarrouk’s medium at an optimal temperature of 35°C and a photosynthetic photon flux density (PPFD) of 100 μmol photons m^-2^s^-1^ supplied by white fluorescent tubes or as otherwise stated. The cell culture was aerated with 0.2 μm filtered air at a flow rate of 1 ml/min. An inoculum at the mid-logarithmic phase of cell growth to an initial inoculum concentration of about 0.166 g/l (OD_560_ 0.1) was dried at 40°C until a constant weight was achieved. The chemical structures of the compounds in the native and thermally treated algal biomasses were studied using an attenuated total reflection Fourier transform infrared spectroscopy (ATRFTIR) spectrometer Hyperion 2000 (Bruker).

### FTIR spectroscopy of Spirulina platensis biomass

After measuring the optical density at 660 nm of the liquid media following 7 days of growth, *S. platensis* samples were transferred to 15-ml glass centrifuge tubes (ELTA 2000, Sofia, Bulgaria) and centrifuged at 1.717 g for 10 min at room temperature. The resulting pellets were washed twice with deionized water to remove residual salts and precipitates. Subsequently, the pellets were resuspended by vortex in deionized water at a ratio of 1 : 3 to concentrate or dilute the sample to an optical density of 1.5 at 660 nm. Five microliters from each mixture were pipetted into the FTIR ATR (MIRacleTM Single Reflectance Attenuated Total Reflectance) sample cell (Hyperion 2000, Bruker). The biomass was dried by nitrogen purge at a rate of 2.0 l/min for 5 min. The sample was then scanned in the FTIR using Happ-Genzel apodization at a wavenumber range of 4000–600 cm^-1^ and a resolution of 4 cm^-1^ for 45 scans. Three spectral measurements were obtained for each of the six samples.

### Bacterial strains and growth conditions

*L. delbrueckii* subsp. *bulgaricus* WDCM 00102 was sourced from the National Bank for Industrial Microorganisms and Cell Cultures in Sofia, Bulgaria. The strain was maintained frozen at −80°C in skim milk (120 g). To revive the cells, they were resuspended in skim milk, and 2% of the rehydrated culture was transferred to MRS broth. The cultures were then grown in MRS broth at 37°C for 24 h. After incubation, the cultures were centrifuged at 4000 rpm for 15 min at 4°C. The supernatant was discarded, and the cells were washed twice with 0.85% NaCl by centrifugation at 4000 rpm for 15 min at 4°C. Finally, the cells were resuspended in a specific cryoprotective medium containing various concentrations of dried *S. platensis* biomass (5, 7.5, 10, 12, 15%).

### Freeze-drying procedure

The cell suspension of *L. delbrueckii* subsp. *bulgaricus* WDCM 00102, mixed with *S. platensis* biomass at concentrations ranging from 5 to 15%, was collected to count the original colony-forming units before freeze-drying. The tubes were then prefrozen at −80°C for 12 h and dried in a freeze dryer (Hochvakuum-TG-16.50) with contact plates heating at a condenser temperature of −60°C and a chamber pressure < 20 Pa for 36 h. For further analysis, the freeze-dried *L. delbrueckii* subsp. *bulgaricus* WDCM 00102 cells were rehydrated in 10 ml of sterile 0.85% NaCl solution, homogenized slightly for 30 s through a vortexer, and the colony-forming units were counted according to Pasteur’s method (O’Sullivan et al., 2010; Rastoll et al., 2013). The survival rate of the cells was expressed as the number of live cells after freeze-drying × 100/live cells before freeze-drying. All experiments were conducted in triplicate. The *L. delbrueckii* subsp. *bulgaricus* WDCM 00102 cells were rehydrated in 10 ml of sterile 0.85% NaCl solution after different storage times at 5 or 25°C (0, 5, 10, 15, 20, and 25 days after the start of the storage experiments). The colony-forming units were determined according to Pasteur’s method or by measuring the pH value using a pH electrode (Milwaukee Ltd, Model MW180 MAX) during their cultivation in a fat-free milk medium (10% dry matter).

### Water activity and water content of lyophilized samples

The water activity of algae-based biomass was measured by drying the freeze-dried samples and using an Aqualab water activity instrument (Stanton Redcroft). The residual water content of *S. platensis* biomass – lyophilized samples containing *L. delbrueckii* – was determined by drying at 105°C for 4 h to obtain completely dried samples on glass plates. The weight of each sample before and after drying was recorded to calculate the water content, which is expressed as the water mass × 100/total powder mass (%).

### Bacterial plate counts

Viable bacterial plate counts were determined before and after the freeze-drying process and after storage. Dried *L. delbrueckii* subsp. *bulgaricus* WDCM 00102 was resuspended in 1 ml of 0.85% w/v sodium chloride solution. Two percent of the rehydrated liquid culture was inoculated into 10 ml of skim milk with a concentration of 4% dry matter. The pH value of the skim milk was monitored during the incubation of the rehydrated *L. delbrueckii* subsp. *bulgaricus* WDCM 00102 strain at 37°C, with readings taken every hour over a 24-h period.

### Reproducibility of the results

Each experiment was conducted with duplicate samples using three independent cultures of bacteria. The relative differences were reproducible regardless of the cultures used. Analysis of variance (ANOVA) was performed on the viable counts and lag times corresponding to the different treatments using Statistix 8 Software (Analytical Software, Florida, USA). Differences were assessed using paired sample *t*-tests, and differences with a *P* –value < 0.05 were considered statistically significant.

## Results

**Fig. 1:**
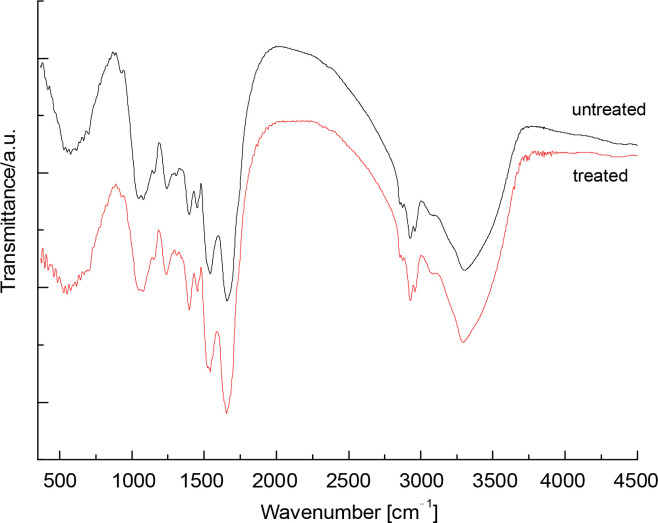
FTIR spectra of the raw (Sp) and thermal (red line) treated *Spirulina platensis* biomass at the wavelength range of 450–4500 nm

FTIR spectra of raw (nonthermally treated) and thermally treated up to 120°C *S. platensis* biomass were obtained in the region of 4500–450 cm^-1^ and are presented in Figure 1. The spectrum of raw *S. platensis* shows signals characteristic of peptide components and polysaccharides. The biomass spectrum of *S. platensis* exhibited a wide band centered at 3302 cm^-1^ related to O–H and N–H groups, and a band in the region of 3000–2750 cm^-1^ assigned to C–H groups. Additionally, the presence of carbonyl groups at 1750 cm^-1^ (esters) and 1659 cm^-1^ (amides) was observed. A vibration at 1543 cm^-1^ was associated with N–H groups. The region from 1500 to 500 cm^-1^ corresponds to overlapping bands, including C–H band deformation and C–O stretching.

For the maltodextrin spectrum, the wide band centered at 3402 cm^-1^ was attributed to hydroxyl groups. Contributions of C–H groups in the range of 3000–2750 cm^-1^ and diverse bands in the region of 1500–500 cm^-1^ were also observed. The band at 1643 cm^-1^ corresponds to water bound to maltodextrin, a typical feature of polysaccharides. The influence of temperature treatment (up to 120°C) on the biomass composition of *Spirulina* was studied. Compared to raw *S. platensis*, a decrease in the intensity of the carboxylate band (1398 cm^-1^) was observed. The C–N and II amide bands also shifted by about 3 cm^-1^ towards longer wavelengths. Additionally, a change in the shape of a short-wave band at 1541 cm^-1^ was visible, related to the decrease in intensity of the signal, which was hidden under the second amide band, originating from the vibrations of asymmetric carboxylate groups.

**Fig. 2:**
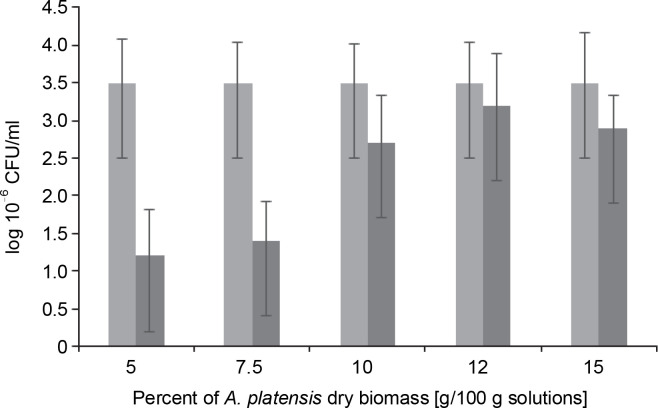
Colony Forming Units as log10 of *Lactobacillus bulgaricus* WDCM 00102 strain recovered after desiccation over *Spirulina platensis* biomass at different concentrations of *Spirulina platensis* biomass in the cryoprotective media; counts of microorganisms before desiccation (control) (■) and desiccated in the presence of *Spirulina platensis* biomass (■)

*L. bulgaricus* WDCM 00102 was dried for an average duration of 48 h in the presence of *S. platensis* biomass at increasing concentrations from 5 to 15%. The efficiency of *S. platensis* biomass in protecting *L. bulgaricus* against these dehydration conditions was then evaluated, and the results are presented in Figure 2. When microorganisms were lyophilized in the presence of a protectant at 5%, there was a decrease of 1.25 CFU logarithms compared to the nondehydrated cells (control). An increase in *S. platensis* biomass content in the range of 5 to 12% led to a proportional increase in colonyforming units of the strain, up to 3.5 CFU, during the vacuum-sublimation drying process. However, this value decreased to 3.0 CFU when the amount of *S. platensis* biomass introduced into the cryoprotection medium increased to 15%.

In fact, under the conditions of the vacuum freeze-drying process in the presence of algae-based biomass as a cryoprotective agent, the plate counts of *L. bulgaricus* WDCM 00102 were only one logarithm lower than those corresponding to the nondehydrated cells. No significant differences (*P* > 0.05) in bacterial recovery were observed between samples when *S. platensis* biomass was used at concentrations of 12 and 15% w/w (Fig. 2). After freeze-drying the bacteria in the absence of *S. platensis* biomass, a threefold decrease in the colony-forming units was observed compared to the control (without algae-based biomass as a cryoprotective agent). This indicates that *L. bulgaricus* is very sensitive to the hydric stress produced by freeze-drying. It can be concluded that after freeze-drying, *S. platensis* biomass at 10 and 12% was a very efficient cryoprotectant in the recovery of *L. bulgaricus*.

To evaluate differences in the protective role of *S. platensis* biomass on the recovery of *L. bulgaricus* after freeze-drying, water activities, and residual water contents after drying, growth kinetics, and survival after storage at different temperatures were compared for all concentrations of *S. platensis* biomass used. The values of the final water activities of the samples are displayed in Table 1. The water activity of the microorganisms freeze-dried in the absence of *S. platensis* biomass was 0.37 ± 0.02, slightly lower than that of the bacteria freeze-dried in the presence of *S. platensis* biomass, with its value increasing from 0.40 ± 0.02 to 0.42 ± 0.03 when changing the *S. platensis* biomass content from 5 to 10%. The increase in the biomass concentration of *S. platensis* in the cryoprotective medium from 10 to 15% was not accompanied by changes in the water activity value (*P* < 0.05). Similarly, the residual water content of samples dried in the absence of *S. platensis* biomass was 0.37 ± 0.02 g/g dry mass and 0.42 ± 0.03 g/g dry mass in the presence of *S. platensis* biomass (10%). This indicates that the presence of *S. platensis* biomass may preserve the level of remnant water in freeze-dried samples, thus protecting the cells.

**Table 1:** Water activities after freeze-drying *L. bulgaricus* WDCM 00102 in the presence of different concentrations of *Spirulina platensis* biomass (%)

Concentrations of *Spirulina platensis* biomass [%]	Water activity [a_w_]
Without GO	37 ± 0.02
5.0	0.40 ± 0.02
7.5	0.41 ± 0.01
10	0.42 ± 0.03
12	0.42 ± 0.01
15	0.42 ± 0.03

**Fig. 3:**
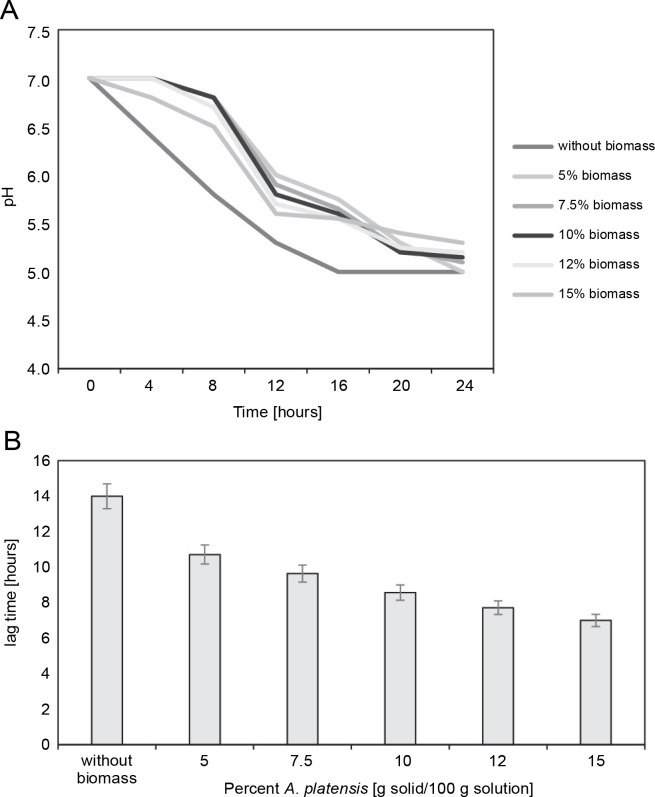
(A) Decreasing of pH-value during cultivation of *L. delbrueckii* subsp. *bulgaricus* WDCM 00102 freeze-dried in different concentrations of *Spirulina platensis* biomass in fat-free milk medium with 10% of dry matter; (B) increase in the lag time with respect to the control (nonfreeze-dried microorganisms) of *L. delbrueckii* subsp. *bulgaricus* WDCM 00102 grown in milk after being freeze-dried in different conditions (*P* < 0.05)

The growth kinetics in the milk of the freeze-dried microorganisms in the presence of *S. platensis* biomass were determined by registering the decrease in pH (Fig. 3A, 3B). Freeze-drying in the absence of *S. platensis* biomass led to a considerable increase in the lag time (lag time: 14 h) (*P* < 0.05), indicating serious bacterial damage.

Freeze-drying in the presence of *S. platensis* biomass at concentrations from 5 to 15% reduced bacterial damage, as revealed by the decrease in the lag times from 12 to 8 hours. The protective effect of *S. platensis* biomass on bacterial damage depended on the concentration of *S. platensis* biomass, with the lowest level of damage observed at 12% addition of *S. platensis* biomass. Increasing the algae biomass content in the cryoprotective media up to 15% led to an insignificant decrease in the value of the lag time of the *L. bulgaricus* WDCM 00102 strain.

Figure 3B depicts the decrease in the lag time for *L. bulgaricus* WDCM 00102 grown in milk after freeze-drying. According to this figure, freeze-drying in the presence of 15% *S. platensis* biomass represented the condition where the lowest bacterial damage was observed (lag time: 6.5 h) (*P* < 0.05). When the strain was freeze-dried in the presence of 10% *S. platensis* biomass, the protective effect decreased considerably (lag time: 9.6 h). The cryoprotective effect of *S. platensis* biomass on the viability of *L. bulgaricus* WDCM 00102 strain was similar for both concentrations analyzed – 10 and 12% (*P* < 0.05).

**Fig. 4:**
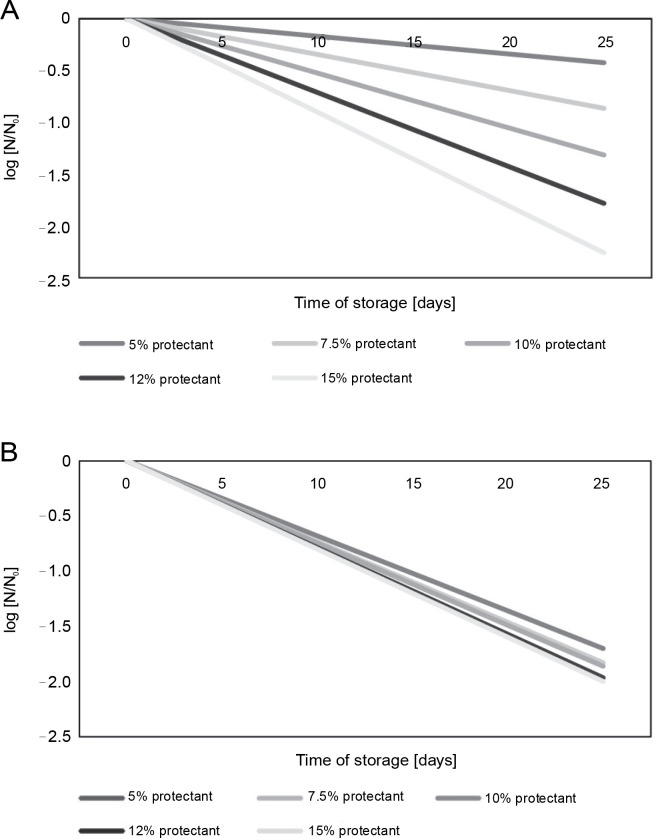
Survival of *L. delbrueckii* subsp. *bulgaricus* WDCM 00102 after freeze-drying in the presence of *Spirulina platensis* biomass and stored at 5 or 25°C; the *N*/*N*_0_ vs time after freeze-drying was plotted for all the conditions assayed; *N* – CFU after storage, *N*_0_ – CFU after freeze-drying and before storage; (A) freeze-drying in the presence of *Spirulina platensis* biomass at 5°C (*P* < 0.05); (B) freeze-drying in the presence of *Spirulina platensis* biomass at 25°C (*P* < 0.05)

Figure 4A and 4B illustrate the decrease in microbial survival after freeze-drying in the presence of *S. platensis* biomass during the storage of freeze-dried *L. bulgaricus* WDCM 00102 strain at 5 and 25°C for 25 days. The loss of viability can be described by simple exponential decay corresponding to first-order kinetics:

log (*N*/*N*_0_) = −*k* × *t*

where *N* – CFU after storage, *N*_0_ – CFU after freeze-drying and before storage, *k* – rate constant of microbial inactivation, *t* – time of storage. The rate constant (*k* ) of microbial inactivation, expressed as the logarithmic value of viable cells at the beginning of storage (*N*_0_) and at a particular storage time (*N* ), changed linearly in relation to the concentration of *S. platensis* biomass used during the storage of freeze-dried *L. bulgaricus* WDCM 00102 strain at temperatures of 5 or 25°C for 25 days. Table 2 summarizes the rate of inactivation (*k*) during storage, obtained from the regression line by plotting the logarithmic value of the residual bacterial count (*N*/*N*_0_) versus the storage period of 25 days. According to these results, *k* was highly dependent on the storage temperature, with low temperatures of 5°C being the most suitable for conservation. The *k* values also indicate that cryoprotection mediated by *S. platensis* biomass at 12% (storage temperature: 5°C) was the best condition for storage.

**Table 2:** Constant viability loss (*k* ) of *L. bulgaricus* WDCM 00102 freeze-dried in the presence of *Spirulina platensis* biomass and stored at different temperatures

Temperature [°C]	Freeze drying conditions				
	*k* (day^-1^) *Spirulina platensis* biomass [g solid/100 g solution]				
	5	7.5	10	12	15
5	0.0170 ± 0.004	0.0176 ± 0,013	0.0180 ± 0.002	0.0187 ± 0.010	0.0190 ± 0.002
25	0,0680 ± 0.005	0.0732 ± 0.010	0.0745 ± 0.012	0.0786 ± 0.015	0.0800 ± 0.010

Additionally, the cryoprotective effect of *S. platensis* biomass at 10 and 12% concentrations on the viability of *L. bulgaricus* WDCM 00102 strain during storage at 5°C was similar and slightly higher than that observed for *S. platensis* biomass at 15% based on the value of the rate constant *k*.

## Discussion

Freeze drying has been widely utilized for the preservation and storage of probiotic lactic acid bacteria. The selection of an appropriate drying medium is crucial to enhance their survival rate during drying and subsequent storage (Oluwatosin et al., 2022). In the present study, the cryotolerance of *L. delbrueckii* subsp. *bulgaricus* WDCM 00102 was improved by adding *S. platensis* biomass at a concentration of 12% in the medium before the freeze-drying process. *Lactobacillus* sp. has demonstrated the highest survival rate of 93.98% when skim milk was used as a cryoprotectant at 12%, attributed to its protein content, which stabilizes cell membrane constituents and prevents cell injury (Kanimozhi and Sukumar, 2023).

An optimal formulation of cryoprotective agents, including 20 g/100 ml skim milk, 3.57 g/100 ml lactose, and 10 g/100 ml sucrose, ensured a cell survival rate of 98% for *L. curvatus* N19 during freeze-drying (Gul et al., 2020). Protein-based and carbohydrate-based matrices have also been employed as cryoprotectants, but their efficacy varied depending on the strain (Gul et al., 2020), as they stabilize cell membrane constituents by creating a protective coating over the cells (Eckert et al., 2018).

The synergistic combination of multiple cryoprotectants can provide greater protective effects than each component separately (Navarta et al., 2011). The use of rapidly penetrating agents prevents both osmotic stress and the formation of extracellular ice (Eckert et al., 2018). Low ice crystal formation was achieved during freezing and frozen storage when binary and multicomponent solutions, including antioxidants (e.g., betaine and sodium glutamate), were included in the protective media (Savedboworn et al., 2019).

The main causes of cell viability loss during freeze-drying include ice formation, and high osmolarity due to a high concentration of internal solutes, which damages membranes, denatures macromolecules, and removes water, affecting the properties of many hydrophilic macromolecules in cells (Barroso et al., 2016; Grattepanche et al., 2013; Ying et al., 2012). To mitigate these adverse changes in freeze-dried cells, several compounds like glucose, rhamnose, mannose, xylose, and galactose have been examined as protective agents in freeze-drying (Barroso et al., 2016; Grattepanche et al., 2013).

Three mechanisms may operate simultaneously during the dehydration–rehydration processes to reduce such adverse changes in the freeze-dried cells. These mechanisms include the direct interaction of the sugar moiety with the polar residues of macromolecules, the formation of glasses in the dried state (vitrification hypothesis), and the exclusion of sugars from the surface (Grattepanche et al., 2013). The latter mechanism of exclusion of sugars from the surface, combined with the values of water activities shown in Table 2, suggests that *S. platensis* biomass may maintain the levels of residual water necessary to preserve cellular structures from damage in freeze-dried microorganisms, allowing for appropriate preservation and avoidance of unnecessary damage.

From a chemical standpoint, *S. platensis* biomass is composed of polyhydroxylated compounds, and their efficiency in bacterial preservation could be explained based on the vitrification and water replacement hypotheses. According to our results, *S. platensis* biomass containing the highest proportion of carbohydrates (13.6%), including glucose, rhamnose, mannose, xylose, and galactose, was the most efficient in protecting cells during desiccation at a 12% concentration (Fig. 2). This efficiency may be attributed to its high water absorption capacity, resulting from the higher content of starch and proteins with polar amino acid residues, making them hydrophilic (Table 1). The slight difference in the protection effect mediated by *S. platensis* biomass at concentrations ranging from 10% to 12% might be due to intrachain H-bond networks. The physicochemical features of the structures of algae-based biomass also promote their interaction with external ions and interchain H-bonding (Fig. 1).

The viability of encapsulated probiotics during storage is influenced by various factors, including storage temperature, type of encapsulating material (carriers, additives like prebiotics, and lyoprotectants), encapsulation process, relative humidity, and oxygen concentration (Carvalho et al., 2004; Fonseca et al., 2003). Temperature is a major factor affecting bacterial survivability during storage (Vivek et al., 2021).

When stored at 4°C, the survival of *L. bulgaricus* WDCM 00102 freeze-dried in the presence of *S. platensis* biomass at a concentration of 12% was high, with the constant of viability loss over 15 days changing in a narrow range of 0.0170 ± 0.004 to 0.0190 ± 0.002. However, increasing the storage temperature to 25°C resulted in a high level of mortality, with the constant of viability loss characterized by the value of 0.0680 ± 0.005 on the 5^th^ day of storage and changing up to the value of 0.0800 ± 0.010.

The higher stability of Bifidobacterium longum HA135, encapsulated in tablets of succinylated-lactoglobulin, at 4°C on storage for 3 months was evaluated in the study of Poulin et al. (2011), while bacterial survival was reduced at storage temperatures of 25°C. For anaerobic bacteria like Bifidobacterium, this temperature impact can be attributed to an oxidation reaction (positive redox potential) in the presence of oxygen. In other words, raising the temperature from 4 to 25°C accelerates oxidation and, as a result, reduces the viability of these anaerobic cells (Poulin et al., 2011).

The synergistic combination of several cryoprotectants can provide higher protective effects than each component separately (Navarta et al., 2011). For instance, *L. plantarum* ATCC8014, *L. paracasei* ML33, and *L. pentosus* ML82, encapsulated with whey permeate alginate-pectin or whey-alginate-pectin (produced by extrusion), exhibited cell viability of > 6 logs CFU/ml after 3 months of storage at 4°C (Eckert et al., 2018). Additionally, alginate beads coated with gelatin were reported to increase the tolerance of *Lactobacillus rhamnosus* ATCC 7469, with the numbers of surviving cells changing from 4.2 × 10^9^ CFU/g to 10^5^ CFU/g after 4 months of storage at 8°C (Lopes et al., 2017).

The rate constant (*k* ) of microbial inactivation, expressed as the logarithmic value of viable cells at the beginning of storage (*N*_0_) and at a particular storage time (*N* ), changed linearly in relation to the concentration of *S. platensis* biomass during storage of freezedried *L. bulgaricus* WDCM 00102 strain at temperatures of 5 or 25°C for 25 days. The *k* values also indicate that cryoprotection mediated by *S. platensis* biomass at 12% (storage temperature: 5°C) was the best condition for storage. Savedboworn et al. (2019) stated that the *k* values during freeze-drying of *L. plantarum* strain were in the range of 1.20 × 10^-2^ to 5.39 × 10^-2^ l/d and 5.79 × 10^-2^ to 2.24 × 10^-1^ l/d during subsequent storage at 4 and 30°C, depending on the concentration of cryoprotectants.

Regarding their capacity to grow in milk after rehydration, *S. platensis* biomass was more efficient at a minimal concentration of 12% in the recovery of *L. bulgaricus* WDCM 00102 after damage and for low-temperature storage. According to Figures 3 and 4, the protective effect of *S. platensis* biomass depended on the concentration. Structural differences between monosaccharide moieties of *S. platensis* carbohydrates play an important role not only in their ability to interact with biomolecules but also in their ability to form glasses where biomolecules are embedded. Further analyses are being carried out to correlate structural and thermodynamic differences between the *S. platensis* biomass preparations studied in this work with their effect on bacterial cryoprotection.

## Conclusion

In this work, we have demonstrated the high efficiency of *S. platensis* biomass at a concentration of 12% in the cryopreservation of *L. bulcaricus* WDCM 00102. Given the physicochemical and nutritional properties of *S. platensis* biomass, its interaction with probiotics could be utilized in the development of commercial synbiotic products. These products could be incorporated into various foods, such as infant formulas and powders containing probiotics in combination with prebiotics, making them valuable functional food ingredients for the manufacture of probiotic foods.
